# Longitudinal observation of OCT imaging is a valuable tool to monitor primary vitreoretinal lymphoma treated with intravitreal injections of methotrexate

**DOI:** 10.1186/s12886-019-1300-1

**Published:** 2020-01-06

**Authors:** Huiying Zhao, Xiaona Wang, Yu Mao, Xiaoyan Peng

**Affiliations:** 1 Beijing Institute of Ophthalmology, Beijing Ophthalmolgy and Visual Science Key Laboratory, Beijing Tongren Eye Center, Beijing Tongren Hospital, Capital Medical University, 17 Hougou Lane, Chongnei Street, Beijing, 100005 People’s Republic of China; 2 Department of Ophthalmology, Beijing Geriatric Hospital, 118 Wenquan Road, Haidian District, Beijing, 100095 People’s Republic of China

**Keywords:** Primary vitreoretinal lymphoma (PVRL), Optical coherence tomography (OCT), Methotrexate

## Abstract

**Background:**

Developing objective and repeatable indicators to evaluate the efficacy of PVRL treatment is important. The quantification of vitreous cells is a traditional criterion; however slight changes are difficult to ascertain. Spectral domain optical coherence tomography (SD-OCT) is objective, repeatable, and easily explained. The purpose of this study is to provide a longitudinal observation of OCT in PVRL treated with intravitreal injections of methotrexate (MTX) and to evaluate the utility of OCT in monitoring responsiveness of PVRL to treatment.

**Methods:**

The medical records of patients with biopsy-positive PVRL attending our hospital between January 2016 and September 2017 who received intravitreal injections of MTX were included in this study. Pre- and post-treatment OCT images were reviewed independently by two researchers.

**Results:**

Of the 24 cases reviewed, 10 patients (18 eyes) were included. SD-OCT abnormalities at the initial visit included vitreous cells (18/18), OR (outer retina) fuzzy borders (12/18), PED (pigment epithelium detachments) (9/18), subretinal hyperreflective infiltration (3/18), intraretinal infiltration (8/18), and SRF (subretinal fluid) (4/18). Post induction treatment, SRF in cases with RD (retinal detachment) was absorbed, and subretinal fibrosis appeared. Other lesions were significantly reduced. Post consolidation treatment, OR fuzzy borders, PED and SRF disappeared in 2 eyes, intraretinal infiltration disappeared in 1 eye, and other abnormalities further improved. Additionally, retinal fibrosis was observed in 3 eyes. One month post maintenance treatment, all abnormalities observed at the first visit vanished. At the last visit, OCT showed subretinal fibrosis and in 3 eyes (16.7%), the disruption of outer retina in 9 eyes (50%) and thinning of the whole layer in 4 eyes (22.2%).

**Conclusions:**

Our observations reveal that characteristic OCT features in PVRL patients can reduce gradually and finally vanish with therapy. We propose that SD-OCT may be employed to monitor the responsiveness of PVRL to treatment, which may influence decision making in the management of this disease.

## Background

PVRL most commonly represents an extranodal presentation of non-Hodgkin’s lymphoma, being of B-lymphocyte origin in 80% of cases [1]. PVRL is associated with primary central nervous system lymphoma (PCNSL) in up to 80% of cases with poor prognosis [1]. Developing objective, unified and repeatable indicators to evaluate the efficacy of PVRL treatment is important. First, for every patient, once the diagnosis has been made, clinicians require reliable means to evaluate disease activity, determine the treatment plan, monitor the treatment efficacy and adjust the treatment regimen according to the treatment effect if necessary [2]. Second, valid guidelines for managing PVRL are not defined [3]. The PVRL regimen varies, even within the same center. PVRL exhibits responsiveness to intravitreal MTX in some patients; however, there is no set regimen, even in a given center [3]. The combination of intravitreal methotrexate and rituximab has also been applied in some centers as a first-line treatment [4]. The use of stem cell transplantation, ibrutinib or intravenous methotrexate for refractory or recurrent cases of PVRL has also been proven to be partially effective in some clinical trials [5]. To arrive at scientifically valid guidelines for managing PVRL, it is necessary to develop objective and uniform indicators to compare the effects of different therapeutic regimens and to understand the effect of choosing different end points of treatment on the prognosis of PVRL.

Nevertheless, an optimal follow-up scheme for treatment options remains to be defined [6]. Traditionally, BCVA and lymphomatous cells in the vitreous are indicators of efficacy evaluation. BCVA is easily available; however, BCVA does not always vary with PVRL therapy, especially when the lesion is located in the peripheral retina. Additionally, during follow-up, visual acuity may decrease due to corneal epithelial injury, secondary cataracts, macular edema and other factors [3]. The amount of vitreal cells can hardly be accurately depicted, and slight changes in the amount of vitreous cells are difficult to ascertain. Currently, the estimation of vitreal cellular load by grading varies by grading system [7]. Limitations of the method include subjectivity, nonlinearity, poor discrimination among the lower levels of haze, and variability, depending on what is actually being graded [8]. Photographic methods are used to grade vitreal cells, which rely on appraising the amount of image degradation present. However, these methods do not directly evaluate cellular infiltrate in the vitreous and can be influenced by light scattering anywhere along the ocular axis [8]. Therefore, studies should be carried out to establish objective, unified and repeatable indicators that can monitor the efficacy of treatment in patients with PVRL.

SD-OCT is useful and essential for detecting and monitoring subretinal and retinal lesions, even in patients with dense vitritis or minimal abnormalities during a fundus examination [9, 10]. SD-OCT is objective, repeatable, and easily explained. In addition, this detection method has been demonstrated as a potential aid to diagnose PVRL [2, 11, 12]. Moreover, SD-OCT demonstrates characteristic hyperreflective foci in the subretinal space or posterior vitreous, hyperreflective infiltration in the inner layers of the retina, sub-RPE deposits, or the undulation of the RPE [2, 11, 12]. Some OCT features, such as hyperreflective foci and subretinal infiltration, are speculated to represent infiltrating lymphoma cells at various levels in the retina [12, 13]. Based on this notion, we speculate that the dynamic observation of the characteristic changes in OCT can help to monitor the responsiveness of tumor cells to treatment. Although there are some existing data, these findings are largely limited to small case series [2, 14] or from different patients [13]. To address this issue, we present a relatively large series of longitudinal SD-OCT images from PVRL patients who were regularly treated with intravitreal injections of MTX.

## Methods

**Fig. 1:**
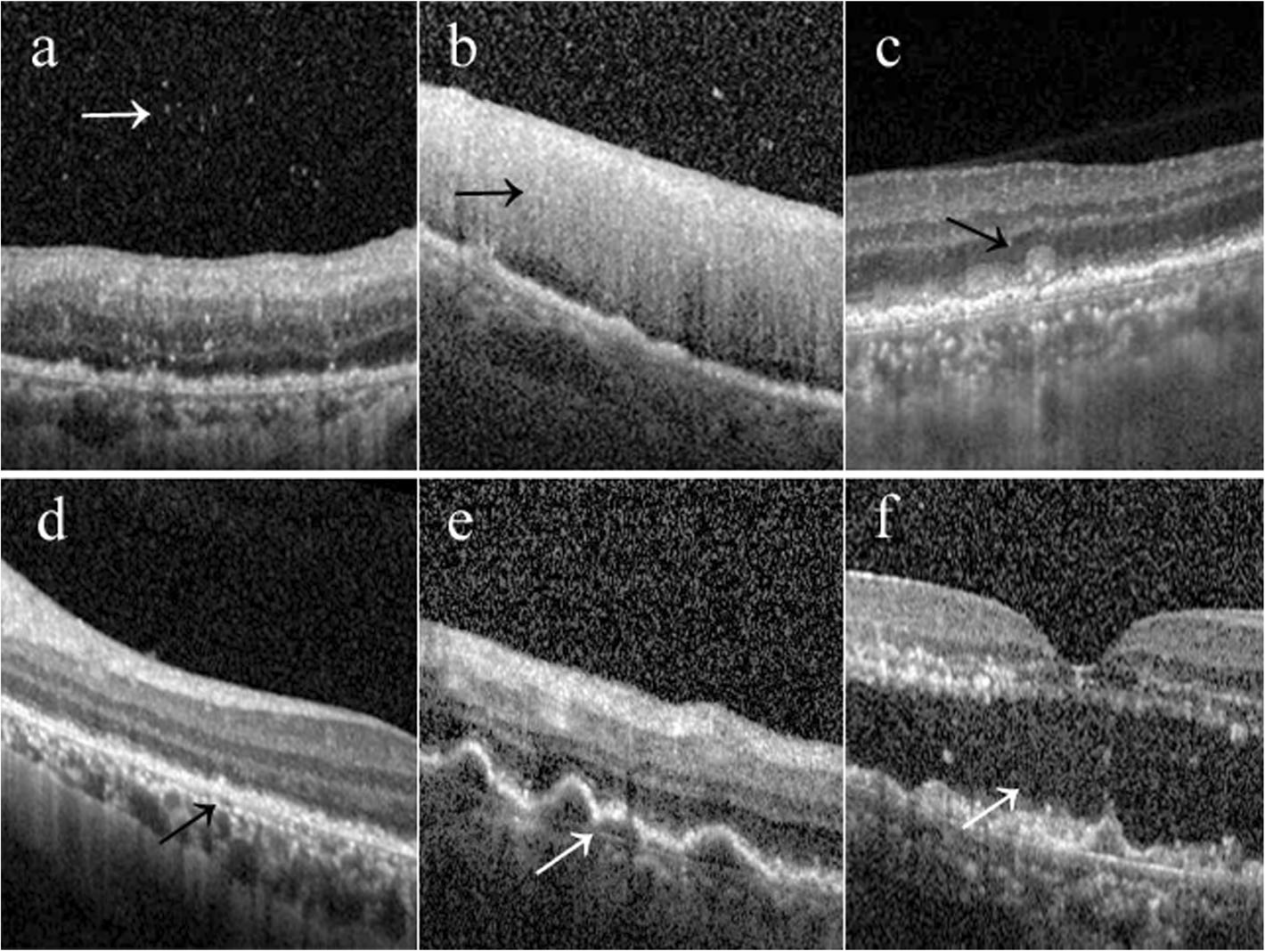
The SD-OCT findings in vitreal retinal lymphoma (arrow). vitreous cells (**a**); intraretinal infiltration (**b**); **c** subretinal hyperreflective infiltration (**c**); OR fuzzy borders (**d**); **e** PED; SRF(**f**)

We reviewed the medical records of patients diagnosed with PVRL between Jan 2016 and Sep 2017 in our hospital. The criteria for a confirmed diagnosis of VRL were based on the cytology of the vitreous biopsy. To be included in the study, the patients should have received intravitreal injections of methotrexate and possess complete ophthalmologic and histopathologic records. The treatment regimen for patients in our series was as follows: an induction phase of twice-weekly intravitreal methotrexate injections for 4 weeks at a dose of 400 μg in 0.1 ml injected at the level of the pars plana using a 30-gauge needle; after induction, weekly consolidation methotrexate injections are given for 1 month; and subsequently, a maintenance phase involves monthly methotrexate injections for half a year. If severe corneal epitheliopathy developed, we stopped the injections for a while. The Heidelberg Spectralis SD-OCT images were depicted using a 30° volume scan pattern with an 8.8 × 8.8 mm scanning area positioned at the center of the fovea. The presence of VRL-related findings in OCT included: vitreous cells; intraretinal infiltration, defined as slightly hyperreflective lesions within the neuroretina; subretinal hyperreflective infiltration; outer retina (OR) fuzzy borders, defined as the blurring of the physiologic external retinal boundaries (i.e., the inner segment/outer segment junction (IS/OS), RPE,external limiting membrane (ELM); PED (pigment epithelium detachments); SRF (subretinal fluid) (Fig. 1). SD-OCT was undertaken pre-treatment, post induction treatment, post consolidation treatment and 1 month post maintenance treatment. The presence of VRL-related findings in OCT was assessed by two independent physicians.

## Results

Of the 24 cases reviewed, 10 patients (5 females and 5 males) with an average age of 57.2 (range, 43–74) years were included. Eight patients (80%) had bilateral involvement. Eighteen eyes were included. The baseline demographics and clinical findings of patients are presented in Table 1. The visual acuity at presentation ranged from light perception to 20/25. Central nervous system involvement presented after the VRL diagnosis in 3 patients (30%).

**Table 1:** Baseline demographics,clinical findings of patients

NO.	Gender	Age	Eye	BCVA at first visit	BCVA at last visit	CNS involvement
1	M	52	OD OS	20/60 20/40	20/20 20/20	no
2	M	74	OD OS	20/400 20/400	20/50 20/30	no
3	F	57	OD OS	20/200 20/30	20/40 20/30	no
4	M	63	OD OS	20/80 LP	20/40 HM	no
5	F	52	OD OS	20/40 20/200	20/30 20/200	after VRL
6	F	52	OD OS	LP 20/25	HM 20/20	no
7	M	64	OS	20/60	20/40	no
8	F	61	OD	HM	20/200	no
9	M	43	OD OS	LP 20/25	HM 20/25	after VRL
10	F	54	OD OS	20/125 20/25	20/40 20/25	after VRL

SD-OCT scans of all involved eyes showed abnormalities at the initial visit. These abnormalities included vitreous cells (18 eyes, 100%), OR fuzzy borders (12 eyes, 66.7%), PED (9 eyes, 50.0%), subretinal hyperreflective infiltration (3 eyes, 16.7%), intraretinal infiltration (8 eyes, 44.4%), and SRF (4 eyes, 22.2%). These patients were regularly followed up during therapy, and the OCT features are listed (Table 2). After induction treatment, SRF in cases with RD (case 4 and case 5) was absorbed, and subretinal fibrosis appeared. Vitreous cells and other lesions were significantly reduced in these cases. After consolidation treatment, OR fuzzy borders, PED and SRF disappeared in 2 eyes, intraretinal infiltration disappeared in 1 eye, and other abnormalities were further improved. Additionally, retinal fibrosis was observed in 3 eyes. At 1 month post maintenance treatment, all abnormalities observed at the first visit vanished. Disruption of the outer retina appeared in 9 eyes. At the last visit, OCT showed subretinal fibrosis and in 3 eyes (16.7%), disruption of the outer retina was observed in 9 eyes (50%) and thinning of the whole layer was observed in 4 eyes (22.2%).

**Table 2:** OCT findings of patients pre- and post- treatment

No	Eye	pre-treatment	post induction treatment	post consolidation treatment	1 month post maintenance treatment	Last visit
1	OD OS	a a	a a	a a	- -	- -
2	OD OS	a,b,e a-c,e,f	a,b,e a,b,e,f	a,b,e a,b,e	i i	i,j i,j
3	OD OS	a-e a-e	a-e a-e	a-e, j a-e	i,j i	i,j i
4	OD OS	a-c a,RD	a-c a-c,e-f	a,b a,g,h	i g,h	i g,h
5	OD OS	a- c,e a,RD	a-c,e a-g	a-c,e a-g,h	i,j g,h	i g,h
6	OD OS	a- f a,b	a- g a,b	a- g,h a,b	g,h,j -	g,h,j -
7	OS	a-e	a-e	a-e	i	i
8	OD	a,b	a,b	a	–	–
9	OD OS	a- c,e a,b	a- c,e a,b	a- c,e a,b	i b	i i
10	OD OS	a a	a a	a a	- -	- -

SD-OCT findings:a vitreous cells, b outer retina (OR) fuzzy borders, c PED, d subretinal hyperreflective infiltration, e intraretinal infiltration, f SRF, g subretinal fibrosis,h retinal fibrosis, i disruption of outer of retina, j thinning of the whole layer ;*RD* retinal detachment

### Representative case report(case 2)

**Fig. 2:**
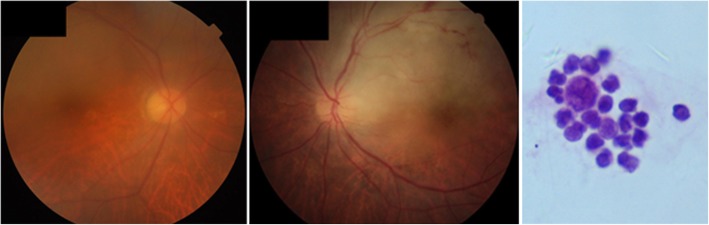
Fundus photography of case 2 at presentation (left and middle). Elevated opacity with an unclear edge in the posterior vitreous in both eyes. In the left eye, opacity was accompanied by superior temporal vein occlusion, patchy hemorrhage and macular edema. Cytology from a diagnostic vitrectomy from the same patient demonstrating large lymphoma cells with large irregular nuclei, prominent nucleoli, and scanty basophilic cytoplasm confirming the diagnosis of vitreoretinal lymphoma (right)

**Fig. 3:**
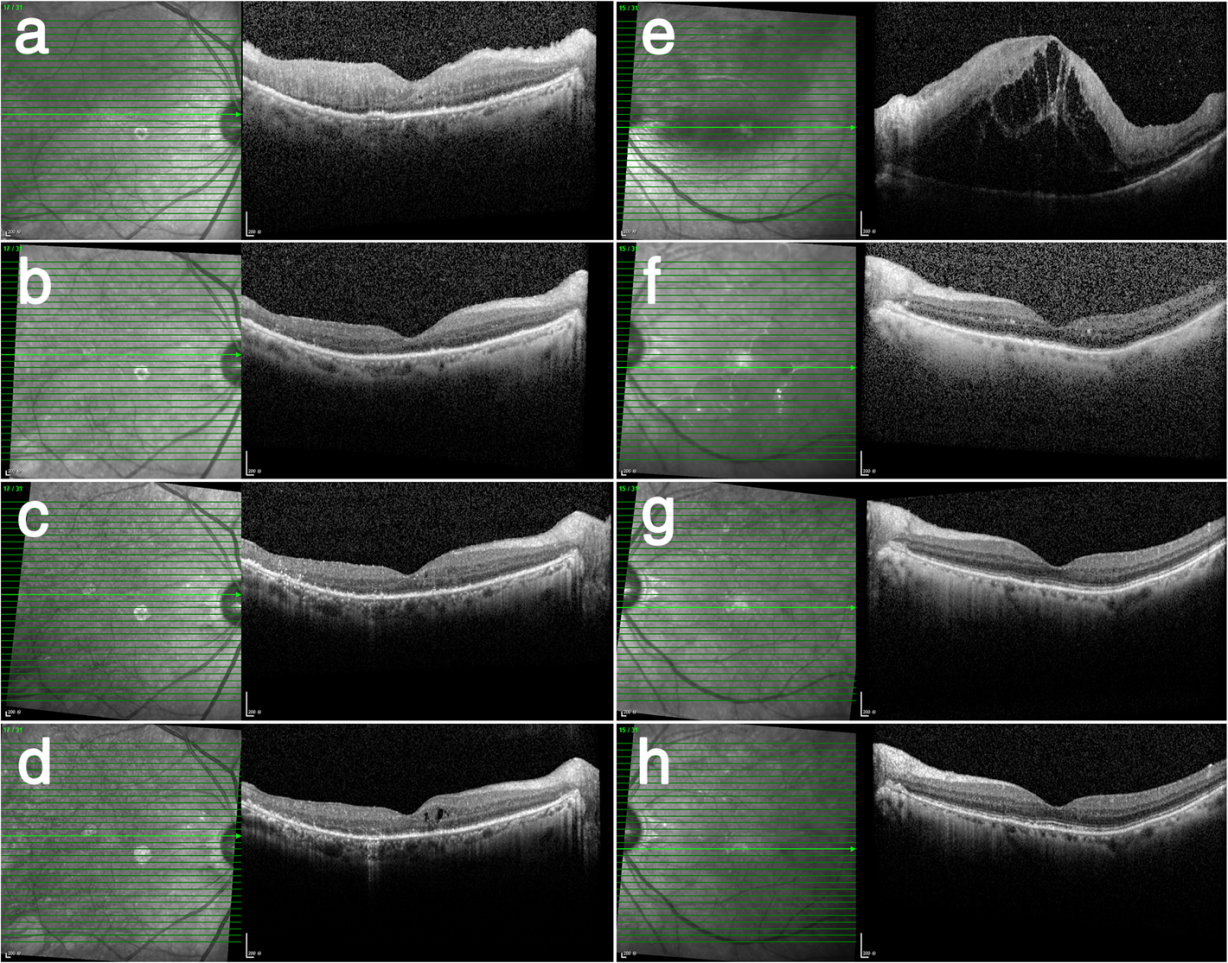
Longitudinal documentation of OCT (case 2). At presentation, OCT revealed vitreous cells, intraretinal infiltration and PED in the left eye (**a**), and vitreous cells, intraretinal infiltration accompanied by macular edema and SRF in left eye (**e**). **b**-**d** revealed changes in OCT of the right eye. After induction treatment, PED disappeared, and the hyperreflective infiltration in all layers of the retina decreased (**b**). After consolidation treatment, hyperreflective infiltration further decreased (**c**). One month after maintenance treatment, the disruption of the outer structure of the retina, atrophy and thinning of the inner retina were revealed (**d**). **f**-**h** revealed changes in OCT of the left eye. After induction treatment, macular edema disappeared, and hyperreflective infiltration in all layers of the retina decreased (**f**). After consolidation treatment, hyperreflective infiltration of the retina further decreased (**g**). One month after maintenance treatment, OCT returned to almost normal (**h**)

A 74-year-old man presented in November 2016, complaining of blurry vision in both eyes for a 9-month duration. The patient was diagnosed with uveitis in March 2016 in another hospital and underwent systemic corticoid therapy for 4 months; however, the results were poor. At presentation, the BCVA in both eyes was 20/400; slit-lamp examination of both eyes revealed a normal anterior segment and sheets of cells in the vitreous. Fundus examination showed elevated opacity with unclear edges in the posterior and multiple subretinal white-yellow lesions in the peripheral retina in both eyes. In the left eye, opacity in the posterior vitreous was accompanied by superior temporal vein occlusion, patchy hemorrhage and macular edema (Fig. 2). OCT examination demonstrated vitreous cells, intraretinal infiltration, PED in both eyes, and macular edema and SRF in the left eye(Fig. 2). A diagnostic vitrectomy of the right eye confirmed the diagnosis of diffuse, large B cell, intraocular lymphoma(Fig. 2). Intravitreal methotrexate was initiated. After the induction phase, the patient’s BCVA improved to 20/100 OD and 20/80 OS. Vitreous cells were reduced. Fundus examination showed that the opacity in the posterior pole disappeared and white-yellow lesions shrank in both eyes. Additionally, in the left eye, retinal hemorrhage and macular edema disappeared. OCT examination demonstrated vitreous cells, intraretinal infiltration, and PED in both eyes reduced (Fig. 3). Nine months later, the patient’s BCVA improved to 20/50 OD and 20/30 OS, the vitreous became clear, and the white-yellow lesions decreased and became pigmented in both eyes. OCT examination demonstrated the disruption of the outer retina(Fig. 3).

## Discussion

PVRL is a disease associated with a coexistent CNS lymphoma in 80% of cases with poor prognosis [1]. Developing objective, unified and repeatable indicators to evaluate the efficacy of treatment for PVRL is important. Traditionally, changes in the amount of vitreous cells or BCVA are common criteria used to determine the response to treatment. However, from month to month, slight changes in the amount of vitreous cells are difficult to ascertain. BCVA does not always vary with changes in PVRL. The need for further information on indicators that can monitor the efficacy of treatment in patients with PVRL is paramount.

In this report, we present the characteristic OCT features of PVRL at the initial visit, derived from a series of SD-OCT images of consecutive eyes with biopsy-proven PVRL. These features were in accordance with previously published reports [2, 11, 12]. We further present that these observed features decreased gradually and finally vanished after therapy. Compared with former previous reports [11, 12, 15], our study obtained a longitudinal OCT images in the same PVRL patient and in a relatively large sample before and after the treatment. This study therefore provides considerable support for the use of SD-OCT as an aid to monitor the responsiveness of PVRL to treatment. We also present that in some eyes, BCVA remained unchanged after treatment, while SD-OCT showed improvement. Therefore, we believe that OCT is superior to BCVA in monitoring PVRL treated with intravitreal injections of MTX.

We confirm that OCT features in patients with PVRL can improve with therapy and finally vanish. In our cases, after the induction phase, all SRF in cases (case 4 and case 5) with RD was absorbed, and other features were significantly reduced. After the consolidation phase, in case 4, OCT abnormalities, except for vitreous cells, disappeared, and OCT abnormalities in other cases further improved. After the maintenance phase, all of the PVRL OCT features vanished. OCT features were speculated to represent infiltrating lymphoma cells at various levels in the retina [11, 12], and indeed, we present further evidence of this phenomenon since these OCT features finally disappeared after the commencement of effective lymphoma-specific therapy.

**Fig. 4:**
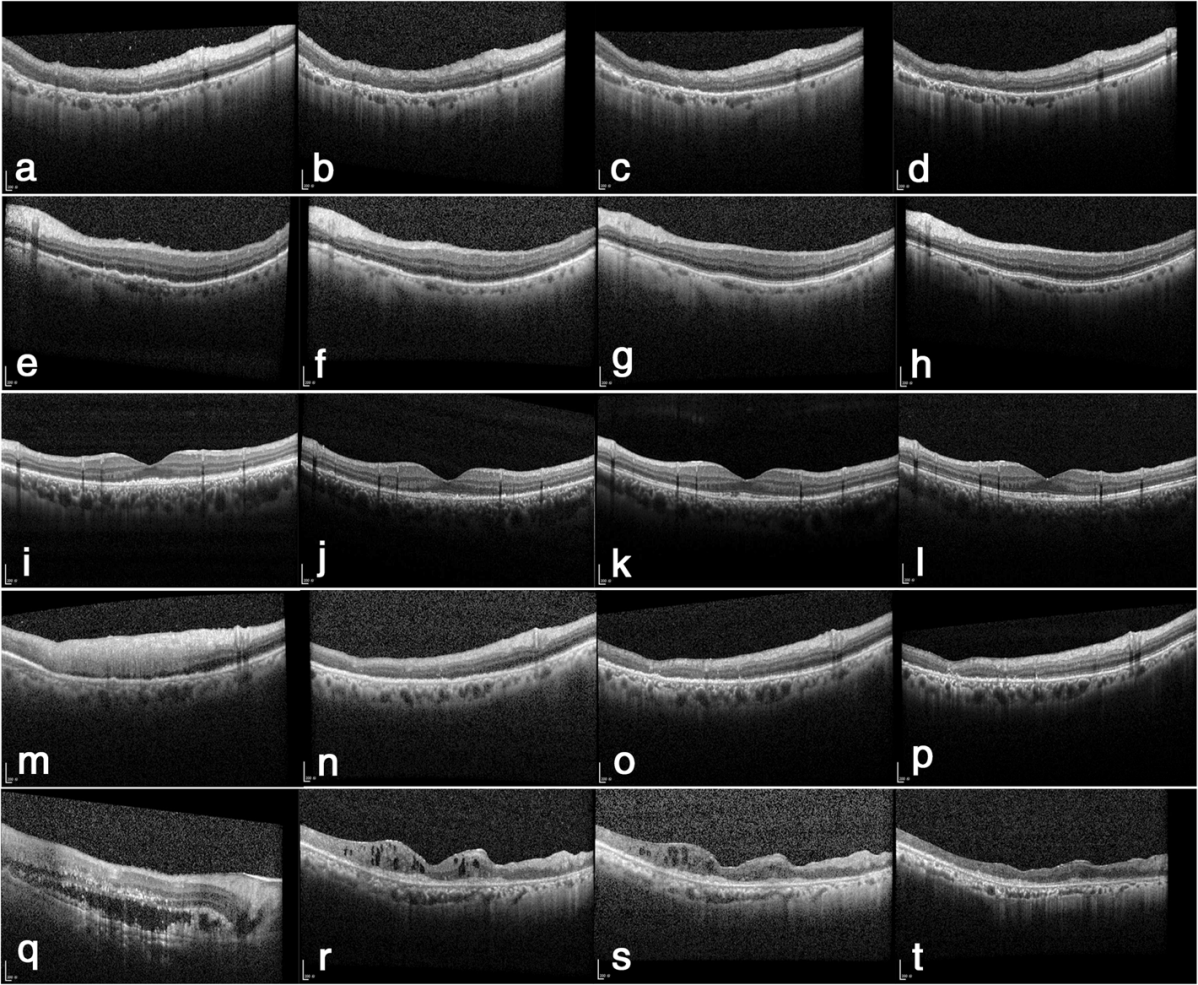
Changes of PVRL features evidenced by SD-OCT. Changes of OR fuzzy borders in OD of case 5 (**a**-**d**). Before treatment, OR fuzzy borders and intraretinal infiltration were observed (**a**). After induction treatment, OR fuzzy borders were significantly reduced (**b**). After consolidation treatment, OR fuzzy borders further reduced (**c**). One month post maintenance treatment, OR fuzzy borders were resolved. In the nasal, the outer retina returned to almost normal, while in the temporal, disruption of the outer retina was observed (**d**). Changes of PED in OS of case 7 (**e**-**h**). Before treatment, PED and OR fuzzy borders were observed (**e**). After induction treatment, PED disappeared (**f**). After consolidation treatment, OR fuzzy borders further reduced (**g**). One month post maintenance treatment, the outer retina returned to normal (**h**). Changes of subretinal infiltration in OS of case 3 (**i**-**l**). Before treatment, subretinal infiltration and OR fuzzy borders were observed (**i**). After induction treatment, subretinal infiltration was absorbed (**j**). After consolidation treatment, OR fuzzy borders further reduced (**k**). One month post maintenance treatment, the outer retina returned to almost normal (**l**). Changes of intraretinal infiltration in OD of case 3 (**m**-**p**). Before treatment, OCT disclosing broad intraretinal hyperreflective infiltration (**m**). Post induction treatment, intraretinal infiltration reduced greatly (**n**). Post consolidation treatment, intraretinal infiltration further reduced (**o**). One month post maintenance treatment, thinning of the inner retina and disruption of outer of retina were observed in the temporal. And other lesions were absorbed and retina returned to normal (**p**). Changes of SRF in the right eye of case 6 (**q**-**t**). Before treatment, OCT disclosing SRF, intraretinal infiltration, subretinal infiltration and OR fuzzy borders (**q**). After induction treatment, SRF was absorbed, subretinal deposits appeared (**r**). After consolidation treatment, hyperreflective infiltration further reduced (**s**). One month post maintenance treatment, atrophy of the retina was observed (**t**)

We believe that the same VRL-OCT features may have different prognoses. In the reported series, some manifestations at, below or above the level of RPE were resolved, with the OCT images returning to normal, while other manifestations were resolved, with the disruption of the interdigitation zone and the ellipsoid zone (Fig. 4). We speculate that the reason for these remaining disruptions was that lymphomatous infiltration contributed to RPE dysfunction, leading to photoreceptor outer segment abnormalities. This finding has also been reported by Keino [15]. In their cases, the most common OCT finding was abnormality of the ellipsoid zone, both at the initial visit and during the follow-up period. In agreement with us, these authors considered the reason for these findings could possibly be that lymphomatous infiltration into the RPE leads to photoreceptor outer segment abnormalities.

Additionally, intraretinal infiltration on OCT can have different outcomes (Fig. 4). Homogeneous lesions are considered to be infiltrating tumor cells that replace the entire layer of the retina [16]. After effective treatment, as shown in the representative case, these hyperreflective lesions gradually diminished, and retina stratification reappeared. Finally, the retina returned to normal or the inner layer atrophied and thinned, and the structure of the outer layers was destroyed (Fig. 4).

SRF can be absorbed rapidly after treatment, and fibrous material deposits can be detected in the outer retina, accompanied by the destruction of the normal structure of the retina (Fig. 4). It is noteworthy that in eyes with exudative retinal detachment at the initial visit, the final BCVA was poor. We believe that the destruction of the retina can be the reason for the poor visual acuity. We recommend exudative retinal detachment as an indicator of poor visual prognosis.

We believe that OCT is superior to BCVA in monitoring PVRL treated treatment with intravitreal injections of MTX. As shown in our cases, BCVA remained unchanged after treatment in 4 eyes (left eyes of cases 3, 5, 9 and 10), although SD-OCT and fundus photography showed marked improvement. In the left eyes of cases 3, 9 and 10, macular fovea was not involved, and the cause of poor BCVA was not PVRL but complicated cataract. Therefore, OCT and fundus photography showed improvement in the tumors outside the fovea, while their BCVA remained unchanged. In the left eye of case 5, although OCT showed that subretinal fluid was absorbed rapidly after treatment, BCVA remained unchanged.

Unfortunately, in the medical record of these cases, the amount of vitreal cells was only recorded as present or absent. We are unable to make an accurate comparison between the effectiveness of therapy based on SD-OCT and that based on vitreous cells under an ophthalmoscope.

Additionally, our study has several other limitations due to its retrospective nature. Our SD-OCT images were limited to the macula, so changes in the peripheral retina and optic disk could not be obtained. The patients should be followed up for a longer time to further confirm whether the patients that were thought to have been cured based on the SD-OCT images were truly cured.

## Conclusions

Our observations reveal that the characteristic OCT features in PVRL patients can reduce gradually and finally vanish with therapy, suggesting that OCT may be employed to monitor the responsiveness of PVRL to treatment, which may influence decision making in the management of the disease. We recommend exudative retinal detachment as an indicator of poor visual prognosis.

## Data Availability

The datasets used during the current study are available from the corresponding author on reasonable request.

## Abbreviations

MTX: Methotrexate
OR fuzzy borders: Outer retina fuzzy borders
PED: Pigment epithelium detachments
PVRL: Primary vitreoretinal lymphoma
RD: Retinal detachment
SD-OCT: Spectral domain optical coherence tomography
SRF: Subretinal fluid
